# Communication With Older Patients With Cancer Using Geriatric Assessment: A Cluster-Randomized Clinical Trial From the National Cancer Institute Community Oncology Research Program

**DOI:** 10.1001/jamaoncol.2019.4728

**Published:** 2019-11-07

**Authors:** Supriya G. Mohile, Ronald M. Epstein, Arti Hurria, Charles E. Heckler, Beverly Canin, Eva Culakova, Paul Duberstein, Nikesha Gilmore, Huiwen Xu, Sandy Plumb, Megan Wells, Lisa M. Lowenstein, Marie A. Flannery, Michelle Janelsins, Allison Magnuson, Kah Poh Loh, Amber S. Kleckner, Karen M. Mustian, Judith O. Hopkins, Jane Jijun Liu, Jodi Geer, Rita Gorawara-Bhat, Gary R. Morrow, William Dale

**Affiliations:** 1 Department of Medicine, University of Rochester, Rochester, New York; 2 University of Rochester Cancer Center National Cancer Institute Community Oncology Research Program Research Base, Rochester, New York; 3 Department of Family Medicine, University of Rochester, Rochester, New York; 4 Department of Medical Oncology, City of Hope National Medical Center, Duarte, California; 5 Department of Surgery, University of Rochester, Rochester, New York; 6 Stakeholders for Care in Oncology and Research for our Elders, Rochester, New York; 7 Department of Health Behavior, Society, and Policy, Rutgers University School of Public Health, Piscataway, New Jersey; 8 Department of Health Services Research, University of Texas MD Anderson Cancer Center, Houston; 9 University of Rochester School of Nursing, Rochester, New York; 10 Novant Health Oncology Specialists, Winston-Salem, North Carolina; 11 Southeast Clinical Oncology Research Consortium National Cancer Institute Community Oncology Research Program, Winston-Salem, North Carolina; 12 Heartland Cancer Research National Cancer Institute Community Oncology Research Program, Decatur, Illinois; 13 Metro Minnesota Community Oncology Research Program, St Louis Park; 14 Department of Medicine, University of Chicago, Chicago, Illinois; 15 Department of Supportive Care, City of Hope National Medical Center, Duarte, California

## Abstract

**Importance:**

Older patients with cancer and their caregivers worry about the effects of cancer treatment on aging-related domains (eg, function and cognition). Quality conversations with oncologists about aging-related concerns could improve patient-centered outcomes. A geriatric assessment (GA) can capture evidence-based aging-related conditions associated with poor clinical outcomes (eg, toxic effects) for older patients with cancer.

**Objective:**

To determine whether providing a GA summary and GA-guided recommendations to oncologists can improve communication about aging-related concerns.

**Design, Setting, and Participants:**

This cluster-randomized clinical trial enrolled 541 participants from 31 community oncology practices within the University of Rochester National Cancer Institute Community Oncology Research Program from October 29, 2014, to April 28, 2017. Patients were aged 70 years or older with an advanced solid malignant tumor or lymphoma who had at least 1 impaired GA domain; patients chose 1 caregiver to participate. The primary outcome was assessed on an intent-to-treat basis.

**Interventions:**

Oncology practices were randomized to receive either a tailored GA summary with recommendations for each enrolled patient (intervention) or alerts only for patients meeting criteria for depression or cognitive impairment (usual care).

**Main Outcomes and Measures:**

The predetermined primary outcome was patient satisfaction with communication about aging-related concerns (modified Health Care Climate Questionnaire [score range, 0-28; higher scores indicate greater satisfaction]), measured after the first oncology visit after the GA. Secondary outcomes included the number of aging-related concerns discussed during the visit (from content analysis of audiorecordings), quality of life (measured with the Functional Assessment of Cancer Therapy scale for patients and the 12-Item Short Form Health Survey for caregivers), and caregiver satisfaction with communication about aging-related patient concerns.

**Results:**

A total of 541 eligible patients (264 women, 276 men, and 1 patient did not provide data; mean [SD] age, 76.6 [5.2] years) and 414 caregivers (310 women, 101 men, and 3 caregivers did not provide data; mean age, 66.5 [12.5] years) were enrolled. Patients in the intervention group were more satisfied after the visit with communication about aging-related concerns (difference in mean score, 1.09 points; 95% CI, 0.05-2.13 points; *P* = .04); satisfaction with communication about aging-related concerns remained higher in the intervention group over 6 months (difference in mean score, 1.10; 95% CI, 0.04-2.16; *P* = .04). There were more aging-related conversations in the intervention group’s visits (difference, 3.59; 95% CI, 2.22-4.95; *P* < .001). Caregivers in the intervention group were more satisfied with communication after the visit (difference, 1.05; 95% CI, 0.12-1.98; *P* = .03). Quality of life outcomes did not differ between groups.

**Conclusions and Relevance:**

Including GA in oncology clinical visits for older adults with advanced cancer improves patient-centered and caregiver-centered communication about aging-related concerns.

**Trial Registration:**

ClinicalTrials.gov identifier: NCT02107443

## Key Points

**Question:**

Does providing a summary of geriatric assessment results and geriatric assessment–guided recommendations to oncologists improve communication about aging-related concerns?

**Findings:**

In this nationwide cluster-randomized clinical trial of 31 community oncology practices that enrolled 541 older patients with advanced cancer, providing a geriatric assessment summary with recommendations to oncologists improved postvisit patient satisfaction and caregiver satisfaction and increased the number of conversations about aging-related concerns. These results were significantly different between the intervention and usual care groups.

**Meaning:**

Integrating geriatric assessment into community oncology care improves patient and caregiver satisfaction and communication about aging-related concerns.

## Teaser

This cluster-randomized clinical trial examines whether providing a geriatric assessment summary and geriatric assessment–guided recommendations to oncologists can improve communication about aging-related concerns.

## Introduction

Patient-centered communication promotes high-quality conversations prioritizing patient and caregiver concerns so that decisions are aligned with their preferences and values. Effective communication is characterized by (1) informed and participatory patients and caregivers; (2) informed, receptive, and patient-centered clinicians; and (3) a health care system providing well-organized and responsive services that are tailored to patients’ and caregivers’ needs.^1,2^ Although studies have demonstrated benefits for interventions that facilitate oncologist-patient communication,^3,4,5^ these interventions were not tailored to address aging-related concerns of older adults receiving cancer treatment and their caregivers.

Older adults represent most patients with advanced cancer seen in community oncology practices.^6,7^ Cancer treatment choices for older adults with aging-related conditions (ie, disability, comorbidity, and geriatric syndromes)^8,9^ are based on extrapolations of evidence derived from clinical trials that enroll younger patients or fit older adults.^10^ Many older adults have unidentified, uncommunicated, and therefore unaddressed aging-related conditions that are associated with morbidity and early mortality.^11^ A communication intervention for oncologists who care primarily for older adults—yet lack aging-related expertise—could improve patient and caregiver satisfaction by bringing attention to often-overlooked aging-related conditions.^12^ Despite controversy,^13^ satisfaction with physician communication is considered a metric for quality of health care and even modest improvements in survey scores are linked to increased reimbursement.^14,15,16,17,18^

To address a “cancer care delivery system in crisis,”^19^^(p1)^ the National Academy of Medicine (formally the Institute of Medicine),^20,21^ the American Society of Clinical Oncology (ASCO),^22^ the Cancer and Aging Research Group,^10,23,24^ and the International Society of Geriatric Oncology,^25^ have all called for improved care delivery that attends to aging-related conditions of older adults with cancer. A key component is geriatric assessment (GA), which uses validated patient-reported and objective measures to capture domains important to older adults such as function (ie, ability to remain independent) and cognition. As highlighted in a recent ASCO guideline,^11^ older adults and caregivers value these GA domains,^26,27^ and GA domains, when formally assessed, influence treatment decision-making.^11,12,28,29,30^ However, aging-related concerns are rarely addressed in oncology care, especially outside specialized academic settings.^12,31,32^

To our knowledge, this study is the first randomized clinical trial evaluating whether GA can meaningfully influence oncology care processes for vulnerable older adults with advanced cancer. With outcome measure selection guided by input from older patients and caregivers,^23,33^ we hypothesized that providing GA information to oncologists would improve patient satisfaction with communication about aging-related concerns by increasing the number and quality of conversations during oncology clinic visits.

## Methods

### Overview

**Figure 1.**
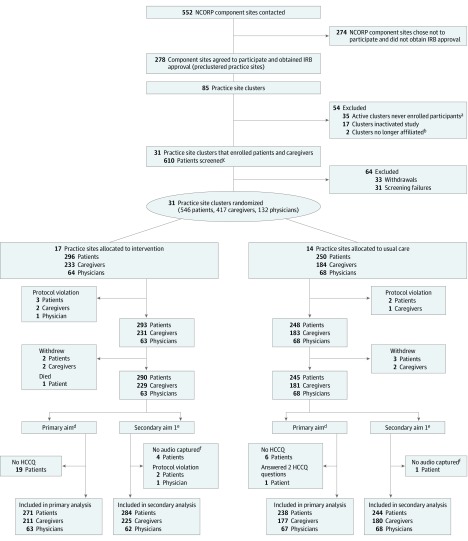
CONSORT Flow Diagram for the COACH (Improving Communication in Older Cancer Patients and Their Caregivers) Trial of Practice Clusters, Oncologists, Patients, and Caregivers Follow-up at 4 to 6 weeks included 472 patients, at 3 months included 410 patients, and at 6 months included 348 patients. Follow-up included 348 caregivers at 4 to 6 weeks, 306 caregivers at 3 months, and 261 caregivers at 6 months. HCCQ indicates Health Care Climate Questionnaire. ^a^Clusters that maintained institutional review board (IRB) approval but never enrolled any participants. ^b^Practices are no longer associated with their respective National Cancer Institute Community Oncology Research Program (NCORP) affiliate or with the University of Rochester NCORP Research Base. ^c^Signed consent and participated in screening process. ^d^Satisfaction with communication about aging-related concerns. ^e^Conversations about aging-related conditions during clinic visit. ^f^Irretrievable, site miscommunication, technical difficulty, or protocol violation.

In this cluster-randomized clinical trial, Improving Communication in Older Cancer Patients and Their Caregivers (COACH), community oncology practices were randomized to the intervention or usual care group (CONSORT diagram in Figure 1 and trial protocol in Supplement 1).^34^ We enrolled participants from October 29, 2014, to April 28, 2017. The University of Rochester and all participating sites obtained approval from their institutional review boards. Participants provided written informed consent.

### Settings and Participants

We recruited community oncology practices within the University of Rochester National Cancer Institute Community Oncology Research Program (NCORP) Research Base network. Oncologists enrolled as participants^12^; only patients of enrolled oncologists were eligible to participate. Other patient eligibility criteria included aged 70 years or older, at least 1 GA domain impairment,^11,25,35,36,37^ an advanced solid tumor or lymphoma, cancer treatment with palliative intent, planned oncology visits for at least 3 months, ability to provide informed consent independently or via a health care proxy, and an understanding of English. Eligible patients chose 1 caregiver aged 21 years or older. Patients with no eligible caregivers could still enroll in the study.

### Study Groups

All patients underwent a GA that evaluated 8 domains—functional status, physical performance, comorbidity, polypharmacy, cognition, nutrition, psychological health, and social support.^11,25,35,36,37^ The GA was mostly patient reported.^37^ Trained coordinators (J.G.) completed the objective performance and cognitive measures. At practices that were randomized to the intervention group, coordinators entered the GA scores into a locked web-based folder (http://www.mycarg.org) that created a tailored GA summary that was printed out for each patient. The summary included information on GA domain impairments and GA-guided recommendations based on literature review,^11^ guidelines,^38^ and expert consensus.^36^ As an example, the summary would include information that a patient recently fell, that falls increase the risk of chemotherapy toxic effects, and a recommendation for physical therapy to prevent falls.^36^ The summary and recommendations were provided to oncologists once prior to an audiorecorded clinic visit. At study entry, oncologists received a brief training about GA and were told that they had autonomy for if and how they wished to use GA for their enrolled patients. For the usual care group, oncologists were alerted only if patients had abnormal scores on depression and cognitive tests.

### Data Collection and Outcome Measures

In both groups, 1 oncology clinic visit within 4 weeks of GA was audiorecorded and transcribed. Within 7 to 14 days of this visit, trained personnel called the patient to assess satisfaction with communication. During the telephone call, the patients completed 2 versions of the Health Care Climate Questionnaire (HCCQ).^39,40^ The first version measures satisfaction with patient-centered physician communication, such as whether the patient feels that the physician understands her or his perspective and encourages participation in decisions (score range, 0-20; higher scores indicate greater satisfaction). Similar to other research,^41^ the second version of the HCCQ modified the language of the questions in the HCCQ to address satisfaction with communication regarding aging-related concerns (HCCQ-age; score range, 0-28); this modified version of the HCCQ was designed with input from advocates who were not enrolled in the trial and was used for the primary outcome (eAppendix in Supplement 2).

A secondary outcome included the number of aging-related concerns discussed at the visit. With experts and 4 coders, a content analysis framework^42^ outlined how to identify aging-related conversations, assess their quality (whether a concern was acknowledged and further explored by the oncologist), and determine whether an acknowledged concern motivated recommendations for specific GA-guided interventions.^3,11,31,32,36,43^ Team coding of the transcribed audiorecordings occurred until interrater reliability^42^ was 70% or greater. Subsequently, for each transcript, coding was performed independently by 2 trained coders, with 20% of transcripts coded by all 4 coders. Final interrater reliability was 82% for number of concerns and 92% for both quality and interventions.

Other secondary outcomes evaluated patient and caregiver quality of life (QoL) as well as caregiver satisfaction with communication. Patients completed the Functional Assessment of Cancer Therapy scale^44^ at enrollment and 4 to 6 weeks, 3 months, and 6 months later. Caregiver QoL was assessed using the 12-Item Short Form Survey^45^ and burden was assessed using the Caregiver Reaction Assessment^46^ at the same time points as patients. Caregivers completed HCCQ surveys that assessed their satisfaction with communication about their concerns related to the patient’s aging-related conditions and overall care (score range for both surveys, 0-20).

### Randomization and Blinding

Accrual records from University of Rochester NCORP studies were used to stratify practice clusters as large or small accruing sites to assure balance in randomization. Randomization was done at the practice cluster level and recruitment of all participants was based on the group to which their practice cluster was assigned. Other than the statisticians, all investigators were blinded to group; blinding was preserved among the telephone team, transcriptionists, and coders.

### Sample Size

Sample size and power considerations were based on the primary aim of the HCCQ-age to address patient satisfaction with communication about aging-related concerns. This design had 80% power at the 0.05 significance level to detect a difference of 1.3 in HCCQ-age scores, with an intraclass correlation coefficient (ICC) of 0.14,^3,32^ corresponding to an effect size of 0.62. Assuming a withdrawal rate of 5% (based on observational cohort data^47^), the targeted accrual was 528 patients. The design had 80% power at the 0.05 significance level to detect a difference of 0.46 in the number of conversations about aging-related concerns, with an ICC of 0.12, corresponding to an effect size of 0.59.^32^ We originally aimed for participation by 16 NCORP practices. Because the recruitment was initially slower than anticipated, we allowed more practices to participate (as specified by the trial protocol in Supplement 1). The total patient sample size did not change.

### Statistical Analysis

Descriptive statistics were used to evaluate demographics, GA results, and clinical information, and bivariate analyses were performed to compare between- group differences in characteristics of patients and caregivers. For the primary outcome, to follow the intent-to-treat principle and to assess the effect of missing values on the study results, we conducted additional analyses including all randomized eligible patients. Under missing at random assumptions, we evaluated the influence of missing data on the study results via multiple imputation.^48^ The examination of the reasons for missing data did not reveal any reason to suspect a missing not at random mechanism. Nevertheless, we also applied sensitivity analysis using pattern mixture models.^49^ Similar to prior research,^50,51^ we conducted responder analyses evaluating the proportion of participants who reported satisfaction scores within a half SD of the HCCQ score from the perfect score; achieving a perfect satisfaction score is commonly advocated as a metric for high quality in practice.^52,53^

Because of the cluster-randomized study design, a linear mixed model method was applied.^54^ The outcome was the response, and the group was the fixed effect. Practices were entered as a random effect independent of residual error. Estimation was performed using restricted maximum likelihood, and the null hypothesis of zero mean difference between groups was tested using an *F* test.^55^ The results are presented as means (or mean difference) adjusted for the practice effect and evaluated as marginal means from the linear mixed model. Practice differences were assessed graphically using best linear unbiased predictors of the mean response for each.

To assess the effect of the intervention on the outcomes over time, we used a longitudinal linear mixed model. An unstructured correlation matrix was used for the repeated measures from the same participant. The model was adjusted for practice cluster using a random effect independent of the within-participant random effects, and it was fit via restricted maximum likelihood.

Every effort was made to facilitate participants’ completion of questionnaires. However, baseline data from some participants were missing, and there was participant withdrawal (Figure 1); anticipating that some patients would not be able to be reached by telephone, the protocol allowed for imputation of the 4- to 6-week HCCQ results to assess the primary aim. Analysis was performed with SAS, version 9.4 (SAS Institute Inc) and R, version 3.5.2 (R Foundation for Statistical Computing) software. All *P* values were from 2-sided tests, and the results were deemed statistically significant at *P* < .05.

## Results

### Participant Characteristics

From October 29, 2014, to April 28, 2017, 31 practice clusters (17 intervention and 14 usual care) enrolled participants, including 131 oncologists, 541 eligible patients, and 414 eligible caregivers (Figure 1). Patients had a mean (SD) age of 76.6 (5.2) years (range, 70-96 years), and 264 (48.8%) were women; most patients had gastrointestinal and lung cancers (278 [51.4%]) and were receiving chemotherapy (369 [68.2%]) (eTable 1 in Supplement 2). There were no essential differences in demographics or clinical characteristics by group. Most patients had 2 or more GA domain impairments (mean [SD], 4.5 [1.5]); the prevalence of GA domain impairments ranged from 93.7% (n = 507) for physical performance to 25.1% (n = 136) for psychological status; 180 patients (33.3%) had possible cognitive impairment. A total of 487 of 541 patients (90.0%) had 3 or more GA domain impairments. More patients in the usual care group had impaired physical performance (239 of 248 [96.4%] vs 268 of 293 [91.5%]; *P* = .03) and social support (82 of 248 [33.1%] vs 74 of 293 [25.3%]; *P* = .05) (eFigure in Supplement 2). Caregivers (n = 414; mean [SD] age, 66.5 [12.5] years; range, 26-92 years) were most likely to be the patient’s spouse or partner (276 [66.7%]; eTable 2 in Supplement 2) and 310 [74.9%] were women. Baseline data for oncologists,^12^ patients,^37,56,57^ and caregivers^37,56,57^ have been published.

### Patient Satisfaction With Communication

**Figure 2.**
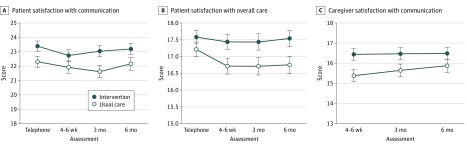
Patient and Caregiver Satisfaction A, Patient satisfaction with communication about aging-related concerns. B, Patient satisfaction with overall care. C, Caregiver satisfaction with communication about the patient’s age-related conditions. Scores were derived using modified versions of the Health Care Climate Questionnaire. The telephone assessment was 7 to 14 days after the audio-recorded clinic visit.

For 509 evaluable patients, the mean (SE) satisfaction score for communication about aging-related concerns was 22.8 (0.27) (range, 5-28 for HCCQ-age) after the clinic visit. The score in the intervention group was 1.09 points higher than in the usual care group (95% CI, 0.05-2.13; *P* = .04; ICC = 0.02). After the clinic visit, the mean (SE) satisfaction score for communication about overall care was 17.4 (0.16) (range, 5-20 for HCCQ). The proportion of patients within a half SD from a perfect score was higher in the intervention group (109 of 271 [40.2%] vs 71 of 238 [29.8%]). Over 6 months, patients in the intervention group were more satisfied with communication about aging-related concerns (difference in mean HCCQ-age score, 1.10; 95% CI, 0.04-2.16; *P* = .04) (Figure 2A) and reported greater satisfaction with overall care (difference in mean HCCQ score, 0.70; 95% CI, 0.06-1.25; *P* = .03) (Figure 2B).

### Number and Quality of Conversations About Aging-Related Concerns

**Figure 3.**
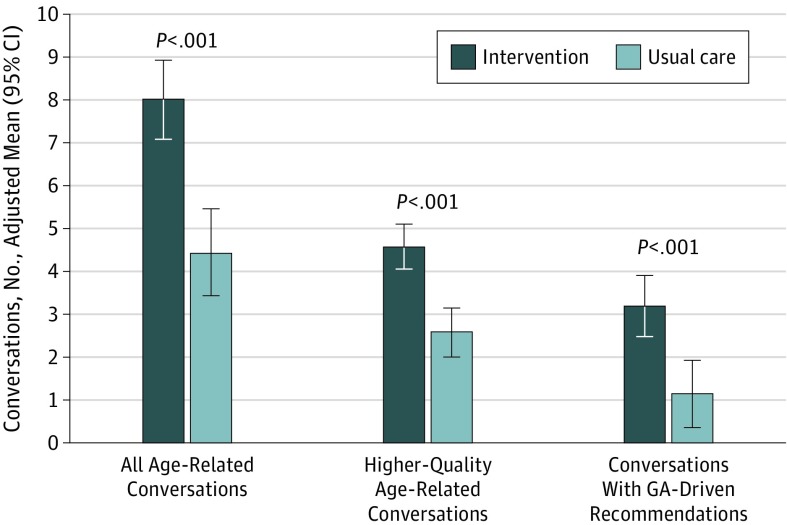
Conversations About Aging-Related Conditions The patient’s visit with the oncologist within 4 weeks of completing the geriatric assessment (GA) was audiorecorded, transcribed, and coded. We used an open coding approach of themes and subthemes to quantify the number of age-related conversations, the number of aging-related discussions with high-quality communication, and the number of conversations of GA-driven recommendations communicated to patients by oncologists.

For 528 evaluable patients, the adjusted mean (SE) number of conversations about aging-related concerns during the oncology clinic visit was 6.34 (0.48) (range, 0-18). There was an adjusted mean of 8.02 conversations in the intervention group compared with 4.43 in usual care (difference, 3.59; 95% CI, 2.22-4.95; *P* < .001; ICC = 0.14; Figure 3). The intervention group had an adjusted mean of 4.60 high-quality conversations, compared with 2.59 in the usual care group (difference, 2.01 [adjusted by practice site]; 95% CI, 1.20-2.77; *P* < .001; ICC = 0.06). There was an adjusted mean of 3.20 conversations about recommendations in the intervention group compared with 1.14 in the usual care group (difference, 2.06; 95% CI, 0.99-3.12; *P* < .001; ICC = 0.30). eTable 3 in Supplement 2 is a joint display^58^ illustrating exemplar quotes with mean conversation numbers by domain.

### Patients’ and Caregivers’ Health-Related Quality of Life

Analyses did not detect any statistically significant differences between groups in Functional Assessment of Cancer Therapy scale score for patients over 6 months (range, 23-108; difference [SE], −0.23 [1.03]; *P* = .82). In addition, there were no differences for caregiver 12-Item Short Form Survey total scores or Caregiver Reaction Assessment subscales.

### Caregiver Satisfaction With Communication

At 4 to 6 weeks after the clinic visit, caregivers in the intervention group were more satisfied with their communication regarding their concerns about the patients’ aging-related conditions (range, 5-20; difference, 1.05; 95% CI, 0.12-1.98; *P* = .03). The proportion of caregivers within a half SD of a perfect score was higher in the intervention group (74 of 189 [39.2%] vs 42 of 158 [26.6%]). Caregivers were more satisfied with their own communication with oncologists with regard to overall care (range, 2-20; difference, 1.34; 95% CI, 0.50-2.18; *P* = .004). The differences in satisfaction scores were not significant when analyzed over 6 months (Figure 2C).

## Discussion

The COACH cluster-randomized clinical trial is the first large multisite intervention study to demonstrate that providing a GA summary with GA-guided recommendations to community oncologists facilitates communication about aging-related concerns and improves patient and caregiver satisfaction with communication and care. COACH enrolled vulnerable older patients with cancer who had significant aging-related conditions—90% had 3 or more GA domain impairments. These patients represent less-fit individuals for whom there is limited evidence for the risks and benefits of cancer treatment,^59^ yet these patients are commonly seen in real-world community practices. Although patients had various cancer types, all were incurable and were treated with palliative intent.

Evidence increasingly supports the use of GA for evaluation and management of older patients with cancer to guide shared decision-making between older patients, caregivers, and oncologists.^11,25^ As highlighted in the ASCO geriatric oncology guidelines^11^ and supported by systematic reviews,^29,60^ GA impairments are associated with chemotherapy toxic effects, lower treatment completion, functional decline, early mortality, and higher health care use. Like others, we found that older patients with a high prevalence of GA domain impairments still receive treatment for advanced cancer, including chemotherapy. Of particular concern is the one-third of patients who had positive screening results for possible cognitive impairment, given the limited evidence for the safety and efficacy of chemotherapy in this group.^61^ The higher prevalence of GA domain impairments compared with other trials reflects our expanded eligibility criteria and our use of a formal GA to evaluate often overlooked aging-related conditions.

Despite patient and caregiver concerns and preferences for maintaining function and cognition,^26,27^ oncologists often do not discuss implications of aging-related conditions or inform older patients and caregivers of heightened risk of adverse events from treatment.^32^ We found that, when GA information was provided, community oncologists used it in communication during the clinic visit, similar to other nongeriatric studies that have systematically provided symptom and QoL information to oncologists.^62,63^ Our results align with this research showing that coordinated care for younger patients that captures patient-reported outcomes improves quality of care and outcomes; for older patients with cancer, personalized care requires attention to aging-related conditions.

We recruited older patients who had several different cancers and treatments, which may have limited our ability to detect QoL effects. In addition, the intervention provided a GA summary during 1 clinic visit only to oncologists; studies that have reported survival and QoL benefits from structured interventions have incorporated evaluation and management of patient-reported outcomes over time^64^ or have used geriatrics-trained professionals.^29,64^ A randomized study of GA-directed therapy for older patients with advanced lung cancer demonstrated reduced toxic effects of treatment and less treatment discontinuation in the GA group owing to improved treatment allocation.^65^ Several ongoing clinical trials will evaluate if GA can help improve clinical outcomes (QoL, toxic effects, and survival) of patients through improved decision-making and GA-guided interventions.^11^

A previous study using baseline COACH data reported that an increasing number of patient GA domain impairments is associated with poor caregiver emotional health and QoL.^37^ Similar to early palliative care models that used specialized nurse coaches to assess and provide management for patients and caregivers, GA-based interventions could be adapted for both patients and caregivers.^66^

### Strengths and Limitations

Strengths of this study include recruitment of a large sample of vulnerable older patients and their caregivers who have rarely been included in cancer trials. This study also demonstrates the ability to conduct multisite trials incorporating GA in the community oncology setting. We attribute our successful completion of the trial in large part to our patient and caregiver research advocate partners from Scoreboard (Stakeholders for Care in Oncology and Research for our Elders) who provided ongoing input and solutions for barriers.^23,33^

Limitations include risk of selection bias, as we enrolled a specific population of older patients; however, these are patients who are commonly seen in community oncology clinics and are underrepresented in research. Although cluster randomization is a strength, since we were testing a model of care as an intervention, there is a risk of selection bias inherent in cluster randomization.^67^ Oncologists in both groups were not blinded, and thus may have modified their discussions of aging; however, the strength of the findings shows that modifying oncologist behavior to increase communication about aging-related concerns is possible.

## Conclusions

To our knowledge, the COACH cluster-randomized clinical trial is the first trial to demonstrate that provision of a formal GA to community oncologists, per ASCO guidelines,^11^ can improve satisfaction and communication for vulnerable older patients with advanced cancer and their caregivers. COACH demonstrated that a practical and convenient GA summary with recommendations for aging-sensitive interventions improves patient-centered outcomes and thus should be considered as the standard of care for older patients with cancer.

## Notes

Supplement 1. **Trial Protocol**

Supplement 2. **eTable 1.** Patient Characteristics by Study Arm **eTable 2.** Caregiver Characteristics by Study Arm **eTable 3.** Average Number of Conversations by Domain, Exemplar Quote, and Oncologist Response **eFigure.** Prevalence of Geriatric Assessment Domain Impairments by Study Arm **eAppendix.** Health Care Climate Questionnaire and Health Care Climate Questionnaire Modified for Aging-Related Concerns

Supplement 3. **Data Sharing Statement**
