# Supplementary material for: Communication With Older Patients With Cancer Using Geriatric Assessment: A Cluster-Randomized Clinical Trial From the National Cancer Institute Community Oncology Research Program
Source: JAMA Oncol. 2019 Nov 7;6(2):196–204. doi: 10.1001/jamaoncol.2019.4728 (PMC6865234; doi:10.1001/jamaoncol.2019.4728)
Supplement: Supplement 2. — eTable 1. Patient Characteristics by Study Arm eTable 2. Caregiver Characteristics by Study Arm eTable 3. Average Number of Conversations by Domain, Exemplar Quote, and Oncologist Response eFigure. Prevalence of Geriatric Assessment Domain Impairments by Study Arm eAppendix. Health Care Climate Questionnaire and Health Care Climate Questionnaire Modified for Aging-Related Concerns [file jamaoncol-6-196-s002.pdf]

Supplementary Online Content

Mohile SG, Epstein RM, Hurria A, et al. Communication with older patients with cancer using geriatric assessment: a cluster-randomized clinical trial from the National Cancer Institute Community Oncology Research Program. *JAMA Oncol.* Published online November 7, 2019. doi:10.1001/jamaoncol.2019.4728

eTable 1. Patient Characteristics by Study Arm
eTable 2. Caregiver Characteristics by Study Arm
eTable 3. Average Number of Conversations by Domain, Exemplar Quote, and Oncologist Response
eFigure. Prevalence of Geriatric Assessment Domain Impairments by Study Arm
eAppendix. Health Care Climate Questionnaire and Health Care Climate Questionnaire Modified for Aging-Related Concerns

This supplementary material has been provided by the authors to give readers additional information about their work.

© 2019 Mohile SG et al. *JAMA Oncology*.

eTable 1. Patient Characteristics by Study Arm

	All patients (N=541)	GA arm (N=293)	Usual care arm (N=248)	*P* value
**Age (mean (SD))**	76.6 (5.2)	76.7 (5.2)	76.4 (5.3)	0.50
70-79	401 (74.1)	220 (75.1)	181 (73.0)	0.73
80-89	127 (23.5)	65 (22.2)	62 (25.0)	
≥90	12 (2.2)	7 (2.4)	5 (2.0)	
**Gender**				0.32
Female	264 (48.8)	137 (46.8)	127 (51.2)	
Male	276 (51.0)	155 (52.9)	121 (48.8)	
**Race/Ethnicity^**				0.68
Non-Hispanic white	482 (89.1)	263 (89.8)	219 (88.3)	
African American	40 (7.4)	19 (6.5)	21 (8.5)	
Others	18 (3.3)	10 (3.4)	8 (3.2)	
**Education**				0.27
<High school	66 (12.2)	36 (12.3)	30 (12.1)	
High school graduate	195 (36.0)	114 (38.9)	81 (32.7)	
Some college or above	279 (51.6)	142 (48.5)	137 (55.2)	
**Income**				0.93
≤$50,000	265 (49.0)	146 (49.8%)	119 (48.0%)	
>$50,000	164 (30.3)	88 (30.0%)	76 (30.6%)	
Decline to answer	109 (20.1)	58 (19.8%)	51 (20.6%)	
**Living arrangements**				0.69
Independent living (more than 1 story)	229 (42.3)	129 (44.0)	100 (40.3)	
Independent living (1 story)	287 (53.0)	151 (51.5)	136 (54.8)	
Others	23 (4.3)	12 (4.1)	11 (4.4)	
**Cancer type**				0.55
Gastrointestinal	138 (25.5)	72 (24.6)	66 (26.6)	
Lung	140 (25.9)	78 (26.6)	62 (25.0)	
Genitourinary	79 (14.6)	46 (15.7)	33 (13.3)	
Breast	69 (12.8)	31 (10.6)	38 (15.3)	
Other	114 (21.1)	65 (22.2)	49 (19.8)	
**Cancer stage**				0.06
Stage III	47 (8.7)	28 (9.6)	19 (7.7)	
Stage IV	480 (88.7)	261 (89.1)	219 (88.3)	
Others	13 (2.4)	3 (1.0)	10 (4.0)	
**Cancer treatment**				
Any treatment (≥1)	529 (97.8)	284 (96.9)	245 (98.8)	0.21
Multiple treatments (≥2)	185 (34.2)	109 (37.2)	76 (30.6)	0.10

© 2019 Mohile SG et al. *JAMA Oncology*.

Chemotherapy	369 (68.2)	205 (70.0)	164 (66.1)	0.28
Monoclonal antibodies	143 (26.4)	94 (32.1)	49 (19.8)	<0.01
Hormonal treatment	97 (17.9)	44 (15.0)	53 (21.4)	0.06
Oral cancer treatment	86 (15.9)	47 (16.0)	39 (15.7)	0.91
Radiation therapy	49 (9.1)	16 (5.5)	33 (13.3)	<0.01
**Enrolled with caregiver**	414 (76.5)	231 (78.8)	183 (73.8)	0.17
**No. impaired by GA domain (mean (SD))**	4.5 (1.5)	4.5 (1.5)	4.4 (1.5)	0.68
Physical performance	507 (93.7)	268 (91.5)	239 (96.4)	0.02
Polypharmacy	453 (83.7)	252 (86.0)	201 (81.0)	0.12
Comorbidity	345 (63.8)	189 (64.5)	156 (62.9)	0.70
Functional status	319 (59.0)	174 (59.4)	145 (58.5)	0.83
Nutrition	326 (60.3)	186 (63.5)	140 (56.5)	0.10
Cognition	180 (33.3)	103 (35.2)	77 (31.0)	0.31
Medical social support	156 (28.8)	74 (25.3)	82 (33.1)	<0.05
Psychological status	136 (25.1)	73 (24.9)	63 (25.4)	0.90

Note: One patient in the GA arm had missing demographic data; ^Race/ethnicity were self-reported by participants

© 2019 Mohile SG et al. *JAMA Oncology*.

eTable 2. Caregiver Characteristics by Study Arm

	All caregivers	GA arm	Usual care arm	*P* value
	**(N=414)**	**(N=231)**	**(N=183)**	
**Age (mean (SD))**	66.5 (12.5)	66.2 (12.5)	66.9 (12.5)	0.57
<70	210 (50.7)	121 (52.4)	89 (48.6)	0.73
70-79	151 (36.5)	81 (35.1)	70 (38.3)	
≥80	50 (12.1)	27 (11.7)	23 (12.6)	
**Gender**				0.32
Female	310 (74.9)	177 (76.6)	133 (72.7)	
Male	101 (24.4)	52 (22.5)	49 (26.8)	
**Education**				0.26
<High school	30 (7.2)	15 (6.5)	15 (8.2)	
High school graduate	118 (28.5)	73 (31.6)	45 (24.6)	
Some college or above	263 (63.5)	141 (61.0)	122 (66.7)	
**Race^**				0.98
Non-Hispanic white	369 (89.1)	206 (89.2)	163 (89.1)	
African American	27 (6.5)	15 (6.5)	12 (6.6)	
Other	15 (3.6)	8 (3.5)	7 (3.8)	
**Relationship**				0.04
Spouse/cohabitating partner	276 (66.7)	151 (65.4)	125 (68.3)	
Son/daughter	94 (22.7)	61 (26.4)	33 (18.0)	
Other	41 (9.9)	17 (7.4)	24 (13.1)	
**Income, annual**				0.54
≤$50,000	151 (36.5)	80 (34.6)	71 (38.8)	
>$50,000	178 (43.0)	100 (43.3)	78 (42.6)	
Decline to answer	81 (19.6)	49 (21.2)	32 (17.5)	
**Living arrangements**				1.00
Independent living (more than 1 story)	188 (45.4)	105 (45.5)	83 (45.4)	
Independent living (1 story)	215 (51.9)	120 (51.9)	95 (51.9)	
Other	7 (1.7)	4 (1.7)	3 (1.6)	
**Comorbidity**				0.88
Yes	162 (39.1)	91 (39.4)	71 (38.8)	
No	249 (60.1)	138 (59.7)	111 (60.7)	
**Anxiety (GAD-7) (≥5)**				0.86
Yes	97 (23.4)	53 (22.9)	44 (24.0)	
No	300 (72.5)	167 (72.3)	133 (72.7)	
**Distress (≥4)**				0.82
Yes	177 (42.8)	98 (42.4)	79 (43.2)	

© 2019 Mohile SG et al. *JAMA Oncology*.

No	230 (55.6)	130 (56.3)	100 (54.6)	
**Depression (PHQ-2) (≥2)**				0.69
Yes	75 (18.1)	40 (17.3)	35 (19.1)	
No	322 (77.8)	180 (77.9)	142 (77.6)	
**SF-12 (mean (SD))**				
Physical health	46.9 (10.5)	47.4 (10.0)	46.3 (11.2)	0.27
Mental health	51.1 (9.8)	50.9 (10.0)	51.2 (9.7)	0.75
Total score	98.0 (14.2)	98.3 (13.7)	97.5 (14.8)	0.55

Note: Three caregivers had missing demographic data

Abbreviations: GAD-7, generalized anxiety disorder 7-item scale; PHQ, patient health questionnaire; SF-12, 12-item short form health survey.

^Race/ethnicity were self-reported by participants

© 2019 Mohile SG et al. *JAMA Oncology*.

eTable 3. Average Number of Conversations by Domain, Exemplar Quote, and Oncologist Response

Geriatric Assessment domain	Average number of conversations			Exemplar quote	Oncologist Response Acknowledged and/or Addressed*
	Intervention arm	Usual care arm	*P* value		
Physical Performance	1.87	0.75	<0.01	D: Okay. So do you feel like you need a cane at least to kind of be sure that you will not fall?<br>P: No.<br>D: Or you don't like that idea.<br>P: No, I don't need that. I'm real stable walking and stuff.<br>D: All right. So when you bend down, you know, things that you may prevent, you know things that may prevent fall will be instead of bending down, you know, bend on your knees rather than stooping from the trunk. All right? So because that will give you more dizziness. So by bending down it doesn’t give you the dizziness. All right?	Addressed; Discussion of assist device and safety recommendations
Functional status	0.58	0.36	<0.01	P: That’s what I pretty much do. I mean there are things that I'd like to do that I am very limited in doing but. . .<br>D: And tell me about some of those.<br>P: Well, basically like housecleaning, scrubbing floors, stuff like that. But there again I can’t really attribute it all to the cancer. I think some of it also has to do with age.	Acknowledged; Exploration of issues related to functional capacity
Comorbidity	0.67	0.41	<0.01	D: Okay. And we’ve already done those things. So, one of the things they want us to talk about is your comorbidities. Your heart condition and your diabetes always put you at bigger risk when you’re taking any chemo pills. We talked about that in the beginning. And that’s some of the reason why we decreased your dose of your chemotherapy. And your other doctors, your cardiologist, your primary care doctor, we send notes to them. So each time I write a note electronically it goes out. So it keeps them informed. And our numbers should be on the notes and you can always take our cards to your primary doctor, your cardiologist just in case they ever have questions for us. So you have a history of diabetes. We are avoiding neurologic agents.	Addressed; care coordination with primary care physician and cardiologist regarding comorbidities, adjusting treatment recommendations based on comorbidities

© 2019 Mohile SG et al. *JAMA Oncology*.

				P: Okay.	
Cognition	0.45	0.12	<0.01	C: And her memory is really a lot worse than it was before.<br>P: Yeah.<br>D: Is that right?<br>C: Yes. And there's times of confusion.<br>D: Okay. [Keyboarding]<br>P: It used to be really sharp.<br>D: I know. We go way back and I know that to be true about you.<br>P: I know it. [Laughs]<br>D: Have you felt that you've been confused? Have you noticed that?<br>P: No, not necessarily myself. But when I answer sometimes I know it's probably not the right answer.<br>D: Have you noticed the memory issues, too?<br>P: Yes. I have. Yeah.<br>D: And do you feel like it's mostly kind of short term stuff? Like why did I come into the room, that kind of thing?<br>P: Exactly.<br>C: Well, that and -<br>P: I don't go looking for stuff, I guess<br>C: Yesterday or last night she could very well have said to me what are we doing tomorrow? Well, we've known for 3 weeks we're coming her.<br>D: Yeah.<br>C: And we talked about it over and over and over again. It just goes away.<br>D: Yeah.<br>P: For the most part it does.<br>C: Hopefully it's no Alzheimer's but we do know.<br>D: Well, yeah, that's a troubling thing. Did you feel like you had any memory issues at all prior to this?	Acknowledged; Exploration of quality and duration of memory problems and concerns
Nutrition	1.71	1.26	<0.01	D: Do you think your appetite could be better? You think you are eating okay?<br>P: It could be a little bit.<br>D: Have you lost any weight? 139.	Addressed; Recommendations for nutritional supplements for weight loss

© 2019 Mohile SG et al. *JAMA Oncology*.

				P: I'm 139.<br>D: And you were 137. So it's gone down and now it's a little up. So it sounds like you are hanging in there. Overall you've lost some weight compared to June. Are you taking any supplements? Do you drink any Ensure or anything like that? Maybe that's something to do, you know, those milk shakes that they have?	
Social Support	0.75	0.22	<0.01	D: Okay? I guess the biggest thing for me is your social support. [Name], right?<br>C: Um hmm.<br>D: [Name (Referring to Caregiver] is a friend.<br>P: Yes.<br>D: Okay. And [Name] is a friend that lives in the neighborhood.<br>P: She lives 80 miles away.<br>D: Okay, not even in the neighborhood.<br>C: No. I live in [Place].<br>D: You live in [Place], okay. So, when we talk in terms of your capabilities you have kids in [state, state and state].<br>P: That's right.<br>D: And none of them are in [state]. You have a friend in [state] but she's 3 counties over.<br>P: Correct.<br>D: So that's my major concern as this process goes forward	Acknowledged; exploration of social support contacts and discussion of risk
Polypharmacy	1.59	1.00	<0.01	D: Medicines. You've got a lot of medicines that you take and do you keep track of them, [Name], pretty well?<br>C: Yes.<br>D: So you know exactly what's . . . and any issues on confusing one pill with another or difficulty with the color of the pills or the shape?<br>C: No. Now I will say at times I'll get them all set up for the week and then I'll go back in there to put them in the little medicine cup to take them to and I'll realize that I neglected to get one in and have to go back and put them in because there are so many sometimes I think I put everything in.<br>D: Okay.	Acknowledged and Addressed; Exploration of safety with pills and recommendation for pill box

© 2019 Mohile SG et al. *JAMA Oncology*.

				C: So I'm not gonna sit here and say I never make mistakes. Over six years you know that's not possible.<br>D: I understand. And you use the little, like rectangular boxes where you put the morning pills in one side and the evening pills in the other side? We can help you get this.	
Psychological Health	0.36	0.11	<0.01	D: So [Name] do you feel depressed?<br>P: At times.<br>D: Okay.<br>P: Not right this minute but, yeah, there's times I feel very depressed.<br>D: Oh, do you feel she's down at times?<br>C: There were certain periods like we all have, you know, lose or new beginnings or whatever where her moods change. Like the loss of her dog not too long ago, you know, and her.<br>D: Okay.<br>C: Dear, dear friend not too long ago. And things like that.<br>D: But no thought of hurting yourself, right?<br>C: Oh no.<br>P: No.<br>D: And you – nobody's hurting you at – where you live? Okay. And do you like to have some counseling?	Acknowledged and Addressed; Exploration of risk for suicide and consideration for counselling

Abbreviations: D=Doctor; P=Patient; C=Caregiver
*Acknowledged=exploration of concern (quality of conversation); Addressed (specific recommendation discussed)

© 2019 Mohile SG et al. *JAMA Oncology*.

eFigure. Prevalence of Geriatric Assessment Domain Impairments by Study Arm

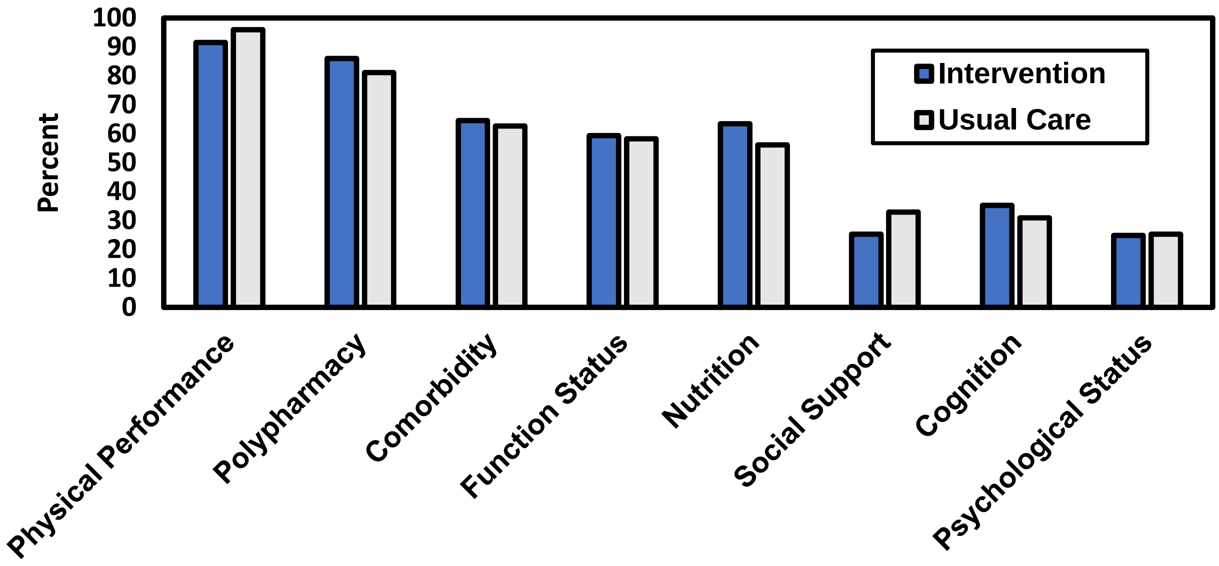

© 2019 Mohile SG et al. *JAMA Oncology*.

eAppendix. Health Care Climate Questionnaire and Health Care Climate Questionnaire Modified for Aging-Related Concerns

5				

Patient ID

Form	Version
**URCC 13070 - COACH**<br>Health Care Climate Questionnaire (HCCQ) (Telephone)	Amd1

Patient Initials

Satisfaction with Overall Communication About Overall Health	Strongly Agree	Agree	Neutral	Disagree	Strongly Disagree
**1.** Your cancer doctor encouraged you to **ask questions**.	☐	☐	☐	☐	☐
**2.** Your cancer doctor was **willing to discuss** any topic of importance to you.	☐	☐	☐	☐	☐
**3.** Your cancer doctor **gave you information** you could understand.	☐	☐	☐	☐	☐
**4.** Your cancer doctor helped you to **feel comfortable** discussing what to expect in the future.	☐	☐	☐	☐	☐
**5.** You feel **understood** by your cancer doctor.	☐	☐	☐	☐	☐

© 2019 Mohile SG et al. *JAMA Oncology*.

5				

Patient ID

Form	Version
**URCC 13070 - COACH**<br>HCCQ-age<br>(Telephone)	Amd1

Patient Initials

Satisfaction with Communication About Other Medical Issues and Aging Concerns	Strongly Agree	Agree	Neutral	Disagree	Strongly Disagree
**1.** Your cancer doctor encouraged you to **ask questions** about your other medical issues in addition to the cancer and/or any health concerns that could be from aging.	☐	☐	☐	☐	☐
**2.** Your cancer doctor was **willing to discuss** your other medical issues in addition to the cancer and/or any health concerns that could be from aging.	☐	☐	☐	☐	☐
**3.** Your cancer doctor **gave you information** you could understand about your other medical issues in addition to the cancer and/or any health concerns that could be from aging.	☐	☐	☐	☐	☐
**4.** Your cancer doctor helped you to **feel comfortable** discussing how cancer treatment could affect your other medical issues in addition to the cancer and/or any health concerns that could be from aging.	☐	☐	☐	☐	☐
**5.** You feel your cancer doctor **understood your overall health**, including your other medical issues in addition to the cancer and/or any health concerns that could be from aging.	☐	☐	☐	☐	☐
**6.** I understand why my cancer doctor suggested my treatment plan because he/she **talked with me about my medical tests** and procedures and how it led to my current diagnosis.	☐	☐	☐	☐	☐
**7.** You feel your cancer doctor **understood you** as a person, including values and beliefs important to you.	☐	☐	☐	☐	☐

© 2019 Mohile SG et al. *JAMA Oncology*.
