# Supplementary material for: Communication With Older Patients With Cancer Using Geriatric Assessment: A Cluster-Randomized Clinical Trial From the National Cancer Institute Community Oncology Research Program
Source: JAMA Oncol. 2019 Nov 7;6(2):196–204. doi: 10.1001/jamaoncol.2019.4728 (PMC6865234; doi:10.1001/jamaoncol.2019.4728)
Supplement: Supplement 3. — Data Sharing Statement [file jamaoncol-6-196-s003.pdf]

Data Sharing Statement

Mohile. Communication With Older Patients With Cancer Using Geriatric Assessment. *JAMA Oncol*. Published November 07, 2019. 10.1001/jamaoncol.2019.4728

Data
Data available: Yes
Data types: Deidentified participant data
How to access data: Supriya Mohile: supriya_mohile@urmc.rochester.edu
When available: With publication

Supporting Documents
Document types: None

Additional Information
Who can access the data: Researchers whose proposed use of the data has been approved
Types of analyses: for any purpose
Mechanisms of data availability: with investigator support, after approval of a proposal, and with a signed data access agreement
Any additional restrictions: This dataset will be available through a repository developed by the Cancer and Aging Research Group: carg.org
